# Integrated Transcriptomic and Proteomic Analyses Reveal Wheat Responses Associated with High-Temperature All-Stage Resistance to *Puccinia striiformis* f. sp. *tritici*

**DOI:** 10.3390/jof12100765

**Published:** 2026-10-11

**Authors:** Chang Su, Xinqian Lyu, Yiyi Tu, Dongli Lu, Guocheng Li, Dongfang Ma, Siwei Zhang, Lujia Yang, Chengyun Li, Yangshan Hu

**Affiliations:** 1 State Key Laboratory for Conservation and Utilization of Bio-Resources in Yunnan, College of Plant Protection, Yunnan Agricultural University, Kunming 650201, China; 2 Ministry of Agriculture and Rural Affairs (MARA) Key Laboratory of Sustainable Crop Production in the Middle Reaches of the Yangtze River (Co-Construction by Ministry and Province), College of Agriculture, Yangtze University, Jingzhou 434025, China; 3 Department of Horticulture, College of Life Sciences, Sichuan Normal University, Chengdu 610101, China

**Keywords:** stripe rust, plant-pathogen interaction, high-temperature all-stage resistance, integrated omics, wheat landrace

## Abstract

The obligate biotrophic fungus *Puccinia striiformis* f. sp. *tritici* (*Pst*) frequently overcomes host immunity through rapid evolution. The wheat landrace Paozimai exhibits high-temperature all-stage resistance (HTAS) to *Pst*, a type of resistance expressed at relatively high temperatures across all growth stages. In this study, we investigated host–pathogen interactions using integrated transcriptomic and data-independent acquisition proteomic analyses. Dual RNA-seq analysis at 192 and 216 h post-inoculation (hpi) revealed a shift in enriched *Pst* pathways during the late latent period, from endoplasmic reticulum protein processing and fatty acid metabolism at 192 hpi to carbohydrate and glutathione metabolism at 216 hpi. In the host, differentially expressed genes (DEGs) were enriched in defense-related signaling pathways at 192 hpi and in metabolic pathways at 216 hpi. Co-expression network analysis identified ribosomal and protein biosynthesis processes that may contribute to defense. Proteomic analysis showed greater overlap across time points, with oxidative phosphorylation enriched at 192 hpi and glycan biosynthesis at 216 hpi. Furthermore, integrated multi-omics and co-expression network analysis identified 16 candidate wheat genes, including genes encoding ribosomal proteins and stress-response proteins. These findings describe temperature-associated host and pathogen responses during HTAS and nominate candidates for functional validation.

## 1. Introduction

Wheat (*Triticum aestivum* L.) is a staple food crop, supporting food security for more than 35% of the population worldwide. However, wheat stripe rust, caused by the obligate biotrophic fungus *Puccinia striiformis* Westend. f. sp. *tritici* (*Pst*), poses a persistent threat to wheat production. This disease is widely distributed across major wheat-growing regions, including China, the United States, and European countries, and can cause yield losses of 5–25% in epidemic years, with extreme cases leading to total crop failure [1,2]. Global surveillance has also documented repeated intercontinental spread of *Pst* and the emergence and onward spread of new clonal lineages, underscoring the transboundary threat posed by this pathogen [3]. The frequent emergence of new *Pst* races with enhanced virulence due to mutation, somatic recombination, long-distance dispersal, and sexual reproduction has resulted in the rapid breakdown of resistance in many commercially cultivated wheat varieties, highlighting the urgency of exploring durable resistance mechanisms and identifying novel resistance resources [4,5]. Temperature is one of the most important environmental factors modulating *Pst* growth, sporulation, and pathogenicity [6]. Global warming can reshape plant–pathogen interactions by altering both pathogen performance and host immunity [7]. Evidence of thermal adaptation in contemporary *Pst* populations further emphasizes the importance of temperature in stripe rust epidemiology [8,9]. Understanding temperature-dependent host resistance is therefore increasingly relevant to sustainable stripe rust management under a warming climate.

Breeding for disease-resistant cultivars is the most environmentally friendly and sustainable strategy for stripe rust control. Wheat resistance to stripe rust is typically classified into all-stage resistance (ASR) and adult-plant resistance (APR) based on the growth stage of expression. ASR is expressed throughout the wheat life cycle but is often conferred by a single race-specific gene, making it prone to loss of effectiveness under pressure from evolving *Pst* populations [10,11]. In contrast, high-temperature all-stage (HTAS) resistance (also referred to as high-temperature seedling-plant resistance) is a unique type of resistance that is stably expressed at elevated temperatures (15–28 °C) or high-temperature induction (20 °C for 24 h at the late latent period) across all growth stages. This type of resistance exhibits a broader spectrum and durability compared to conventional ASR, as its expression is less dependent on specific *Pst* races and more on environmental temperature regulation [6,12]. For high-temperature adult-plant (HTAP) resistance, which is expressed in adult plants under high temperatures, several associated genes such as *Yr18*, *Yr36*, and *Yr46* have been identified [13,14,15], while the high-temperature resistance genes have been shown to participate in pathways including hypersensitive response, reactive oxygen species accumulation, and hormone signal transduction [16,17,18,19,20]. Unlike HTAP resistance, which is expressed at the adult-plant stage, HTAS is expressed from the seedling stage under elevated temperatures and may involve distinct regulatory mechanisms [12]. However, the comprehensive molecular mechanisms underlying HTAS resistance, particularly the dynamic transcriptomic and proteomic networks in response to *Pst* infection under high-temperature conditions, remain largely unclear.

Wheat landraces represent a valuable genetic resource for mining novel resistance genes, as they have evolved adaptive traits to cope with biotic and abiotic stresses through long-term natural selection [21,22]. Paozimai, a traditional Chinese wheat landrace, has been observed to exhibit stable HTAS to multiple *Pst* races under high-temperature conditions [12], making it a useful resource for dissecting the molecular basis of this resistance type. However, the underlying molecular regulatory networks remain largely unresolved. Dual RNA-seq of infected leaves enables the transcriptional responses of wheat and *Pst* to be examined in parallel, while data-independent acquisition (DIA) proteomics measures changes in host protein abundance. Integrating these datasets can reveal host responses detected at one or both molecular levels and help prioritize defense-associated candidate genes [23]. Previous studies have successfully used this integrated strategy to identify key genes and pathways involved in plant-pathogen interactions, such as maize responding to *Fusarium verticillioides* infection via activated phenylpropanoid biosynthesis and wheat enhancing resistance to *Fusarium pseudograminearum* through the TaHDA9-TaP5CS1 module-mediated deacetylation and proline accumulation [24,25]. In wheat stripe rust, integrated transcriptomic and proteomic profiling has identified defense-associated co-expression modules and candidate proteins [26]. Combined transcriptomic and metabolomic analyses have implicated melatonin biosynthesis in wheat defense against *Pst* [27]. In studies of wheat HTAS resistance to *Pst*, transcriptomic analyses have identified differentially expressed genes (DEGs), including those encoding defense-related proteins and transcription factors [28], but proteomic changes and their coordination with transcriptional regulation have not been systematically investigated.

In this study, we aimed to elucidate the molecular mechanisms associated with HTAS in Paozimai and the responses of the host and pathogen through an integrated transcriptomic and proteomic analysis. By comparing wheat transcript and protein abundance together with *Pst* gene expression under normal- and high-temperature conditions at 192 and 216 hpi, we sought to identify pathways and candidate wheat genes associated with this resistance phenotype and to assess the potential contributions of post-transcriptional regulation. These results provide new insights into the molecular basis of HTAS resistance to *Pst* and lay a foundation for the utilization of Paozimai in disease resistance breeding of wheat.

## 2. Materials and Methods

### 2.1. Wheat Planting, Pst Inoculation, and Temperature Induction

Wheat landrace Paozimai was obtained from the Provincial Key Laboratory of Plant Breeding and Genetics, Sichuan Agricultural University. The *Pst* race CYR32 was provided by the Institute of Plant Pathology, Northwest A&F University, and was further multiplied in our laboratory. Wheat seedlings were grown, inoculated with *Pst*, and maintained following the previously published protocols [28]. In brief, Paozimai seedlings were inoculated by brushing their leaves with a suspension of CYR32 urediniospores in sterile water (1:8, *v*/*v*) at the two-leaf stage. After inoculation, the seedlings were kept in the dark at 10 °C and 80% relative humidity for 24 h before being transferred to the designated temperature conditions. To investigate the role of genes involved in HTAS to stripe rust, this study analyzed the dynamic transcriptional changes and protein expression profiles under the following conditions: the 0 hpi sample represents the control group (CK); other samples included those subjected to continuous normal-temperature (NT) treatment at 15/12 °C day/night and sampled at 192 and 216 hpi (N192 and N216), and continuous high-temperature (HT) treatment at 28/15 °C day/night and sampled at 192 and 216 hpi (H192 and H216). The sampling time points were selected based on previous studies of HTAS resistance [12,16,20,29], with the aim of characterizing temporal changes in host molecular responses during the late latent period of stripe rust. The experiment was conducted using a completely randomized design. Three independent biological replicates were performed for each treatment in the RNA-seq experiment. Similarly, three biological replicates were included for each group in the DIA-proteomics analysis. Each replicate consisted of pooled leaf tissue from three different plants.

### 2.2. Transcriptome Sequencing and Identification of DEGs

Total RNA from all samples was submitted to Gene Denovo Biotechnology Co. (Guangzhou, China) for RNA-seq and primary data analysis. Briefly, total RNA was extracted separately from wheat leaves of each biological replicate using the Trizol Kit (Promega Corporation, Madison, WI, USA) according to the manufacturer’s instructions. Poly(A) mRNA was then isolated using oligo-dT beads (Qiagen, Hilden, Germany) to remove residual rRNA. The mRNA was fragmented into short pieces (~200 nt) using a fragmentation buffer, and first-strand cDNA was synthesized using random primers. The cDNA was subsequently purified, end-repaired, adapter-ligated, and enriched by PCR to construct the final sequencing library. Library sequencing was performed on an Illumina NovaSeq X Plus (Illumina, Inc., San Diego, CA, USA).

Clean reads were obtained by filtering raw RNA-seq data and then aligned to the wheat reference genome using Bowtie2 v2.2.8 and HISAT2 v2.1.0 with default parameters. Transcript abundance was calculated as transcripts per million (TPM) for further analysis [30]. Differential expression analysis was performed using the DESeq2 package in R [31], with the statistical model based on a negative binomial generalized linear model and the Wald test. Significant DEGs were screened with the criteria of |log2(fold change)| ≥ 1 and false discovery rate (FDR) < 0.05, followed by Gene Ontology (GO) and Kyoto Encyclopedia of Genes and Genomes (KEGG) pathway enrichment analyses [32]. For *Pst* transcriptome analysis, paired-end reads from infected samples were first aligned to the PST-78 reference assembly using HISAT2. Gene-level counts were obtained from the corresponding GFF annotation using featureCounts v2.1.1. Samples with fewer than 1000 total assigned fungal reads were excluded before differential expression analysis. DEGs were identified using DESeq2, with the criteria of |log2(fold change)| ≥ 1 and FDR < 0.05. Throughout this study, N192 vs. H192 and N216 vs. H216 denote changes under high temperature relative to normal temperature at the corresponding time point.

### 2.3. Weighted Gene Co-Expression Network Analysis (WGCNA)

Using the TPM values of all genes and sample data as input, the WGCNA package in R software v3.6.1 was used to identify modules of highly correlated genes [33] after filtering out genes with low expression. Specifically, genes with TPM ≥ 10 across all samples and a coefficient of variation (CV) > 0.1 were retained, yielding 21,767 genes for subsequent WGCNA. The parameters were set as follows: TOMtype = “signed”, minModuleSize = 50, and mergeCutHeight = 0.25. Modules with a similarity greater than 75% were merged, and genes with similar expression patterns were assigned to the same module. Module eigengenes (MEs), defined as the first principal component of the expression matrix within a given module, were calculated to summarize gene expression profiles [32]. Based on the ME values, modules with high expression under the H216 treatment were screened for functional enrichment analysis.

### 2.4. Data-Independent Acquisition Proteomics and Identification of DEPs

Proteomic analysis was performed on the same leaf samples used for RNA-seq by DIA technology. Proteins were extracted from powdered leaf tissue using a lysis buffer containing 7 M urea, 2% SDS, 0.1% PMSF, and 65 mM DTT. The homogenate was sonicated for 15 min and then centrifuged at 14,000 rpm for 30 min at 4 °C. The supernatant was collected, and the protein concentration was determined using a bicinchoninic acid (BCA) assay [34]. For digestion, 50 μg of protein was reduced with 1 mM dithiothreitol at 55 °C for 1 h, alkylated in the dark with 5 mM iodoacetamide at room temperature for 1 h, and then precipitated with 300 μL of pre-chilled acetone at −20 °C for 2 h. According to the operating instructions, trypsin (Promega, USA) was added to digest the protein precipitate overnight.

All peptide mixtures were reconstituted in buffer A (20 mM ammonium formate in water, pH adjusted to 10.0 with ammonium hydroxide) and then fractionated using an Ultimate 3000 system (Thermo Fisher Scientific, Waltham, MA, USA) coupled with a reversed-phase column (XBridge C18 column, 4.6 mm × 250 mm, 5 μm; Waters Corporation, Milford, MA, USA) under high-pH conditions. The fractionated peptides were desalted, lyophilized, dissolved in solvent A (0.1% formic acid in water), and analyzed using an EASY-nLC1200 system coupled online to an Orbitrap Lumos mass spectrometer (Thermo Fisher Scientific, USA) equipped with a nano-electrospray ion source.

DIA data were processed using Spectronaut 20 with default parameters (BGS Factory Settings). The sequence database was constructed from the protein sequences of the IWGSC RefSeq v2.1 annotation (available at https://urgi.versailles.inrae.fr/download/iwgsc/IWGSC_RefSeq_Annotations/v2.1/, accessed on 10 December 2025). Carbamidomethylation (C, 57.02) was set as a fixed modification, and oxidation (M, 15.99) and acetylation (protein N-term, 42.01 Da) were set as variable modifications. Protein identification was performed using a 1.0% FDR threshold at the precursor, peptide, and protein levels. Automatic calibration was conducted in Spectronaut, and the data were normalized using a local normalization strategy. Peptides passing a 1.0% FDR threshold were retained for protein group quantification using the MaxLFQ algorithm. DEPs were defined as proteins with FC > 1.2 or FC < 0.833 and *p* < 0.05 with *p* values obtained from Student’s *t*-test.

### 2.5. Protein–Protein Interaction (PPI) Network Construction

The Search Tool for the Retrieval of Interacting Genes/Proteins (STRING) database Version 10.5 was employed to analyze the protein–protein interactions (PPIs) among the DEPs identified in this study and to construct corresponding PPI networks [35]. The minimum required interaction score was set to a medium confidence level. The results were visualized using Cytoscape software version 3.6.1 [36].

### 2.6. Statistical Analysis

All statistical analyses were performed using R software (version 3.6.1; https://www.R-project.org/, accessed on 22 December 2025). Graphs were generated using the ggplot2, pheatmap, and WGCNA R packages. Between-group differences were assessed using the Kruskal–Wallis H test and the Wilcoxon signed-rank test, as appropriate. Multiple testing correction was performed using the FDR. A *p* value < 0.05 was considered statistically significant.

## 3. Results

### 3.1. Transcriptomic Profiling of Pst Reveals Dynamic Gene Expression Changes Under High Temperature

**Figure 1:**
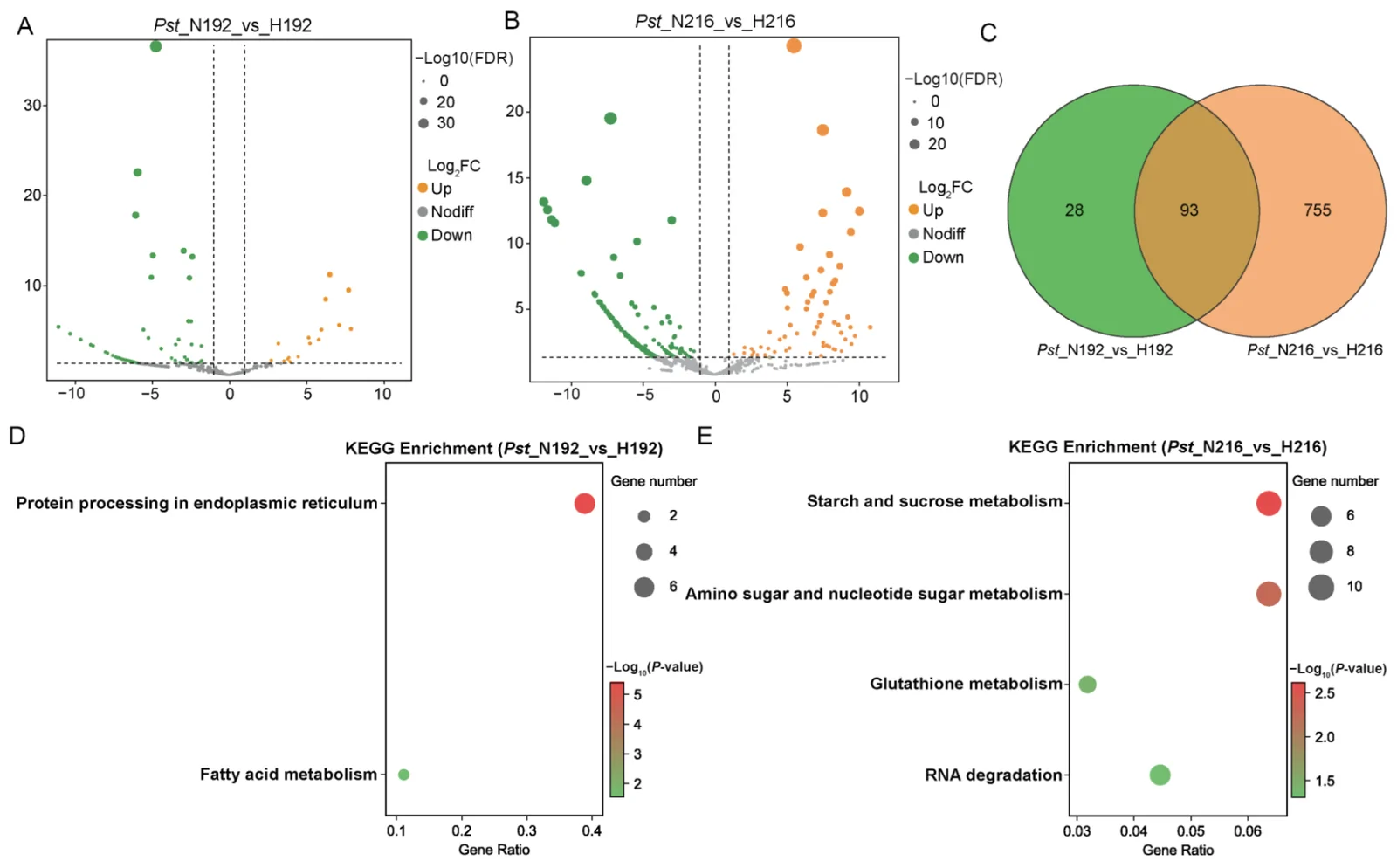
Transcriptome analysis of *Pst* at 192 and 216 hpi under high temperature. (**A**) Volcano plots of differential gene expression for N192 vs. H192 and (**B**) N216 vs. H216. Up- and down-regulated genes are labeled with orange and green dots, respectively. (**C**) Venn diagram of DEGs shared between the two comparisons. (**D**) KEGG enrichment of up-regulated DEGs for N192 vs. H192 and (**E**) N216 vs. H216. The bubble plots show the enrichment of KEGG pathways with *p*-value < 0.05. The abscissa represents the enrichment ratio, and the ordinate denotes the KEGG pathway. The size of bubbles indicates the number of genes annotated to a certain KEGG pathway, and the color represents the *p*-value.

We first focused on the dynamic expression profiles of *Pst* during infection under NT and HT conditions. Based on alignment analysis against the *Pst* genome, a total of 8706 fungal genes were identified as expressed, representing 42.97% of the 20,260 gene models in the PST-78 reference annotation. Subsequently, differential expression analysis revealed that in the *Pst*_N192 vs. H192 comparison, 121 differentially expressed genes (DEGs) were identified, among which 105 were down-regulated and 16 were up-regulated (Figure 1A). In the *Pst*_N216 vs. H216 comparison, 848 DEGs were identified, including 780 down-regulated and 68 up-regulated genes (Figure 1B). Venn diagram analysis indicated that 93 DEGs were shared between the two comparison groups, exhibiting persistent transcriptional alterations in response to high temperature (Figure 1C). Further functional enrichment analysis indicated that the DEGs of *Pst* at 192 hpi were mainly enriched in protein processing in the endoplasmic reticulum and fatty acid metabolism pathways (Figure 1D). At 216 hpi, the DEGs were predominantly enriched in pathways related to starch and sucrose metabolism, amino sugar and nucleotide sugar metabolism, glutathione metabolism, and RNA degradation (Figure 1E). These results indicated temperature-associated transcriptional changes in *Pst* at different infection stages. The regulatory patterns shifted as infection progressed, and the metabolic pathway responses were redirected towards carbohydrate metabolism and precursor metabolism for cell wall component synthesis, accompanied by the involvement of RNA degradation.

### 3.2. Host Transcriptomic Analysis of DEGs and Functional Enrichment

Complementary to the above pathogen-derived transcriptional alterations, host wheat plants exhibit distinct phenotypic and histological differences in response to *Pst* infection under varying temperature conditions. Therefore, we further characterized the host transcriptomic responses of wheat landrace Paozimai to explore the molecular basis of HTAS. After quality filtering, we obtained an average of 6.63 Gb of clean reads per sample. After mapping to the reference genome, a total of approximately 1.2 × 10^5^ genes were identified. Q30 values ranged from 93.0% to 94.7%, the average GC content was 51.99%, and 90.4% of reads mapped to the reference genome (Table S1), indicating that the sequencing data met the quality criteria for downstream analysis.

**Figure 2:**
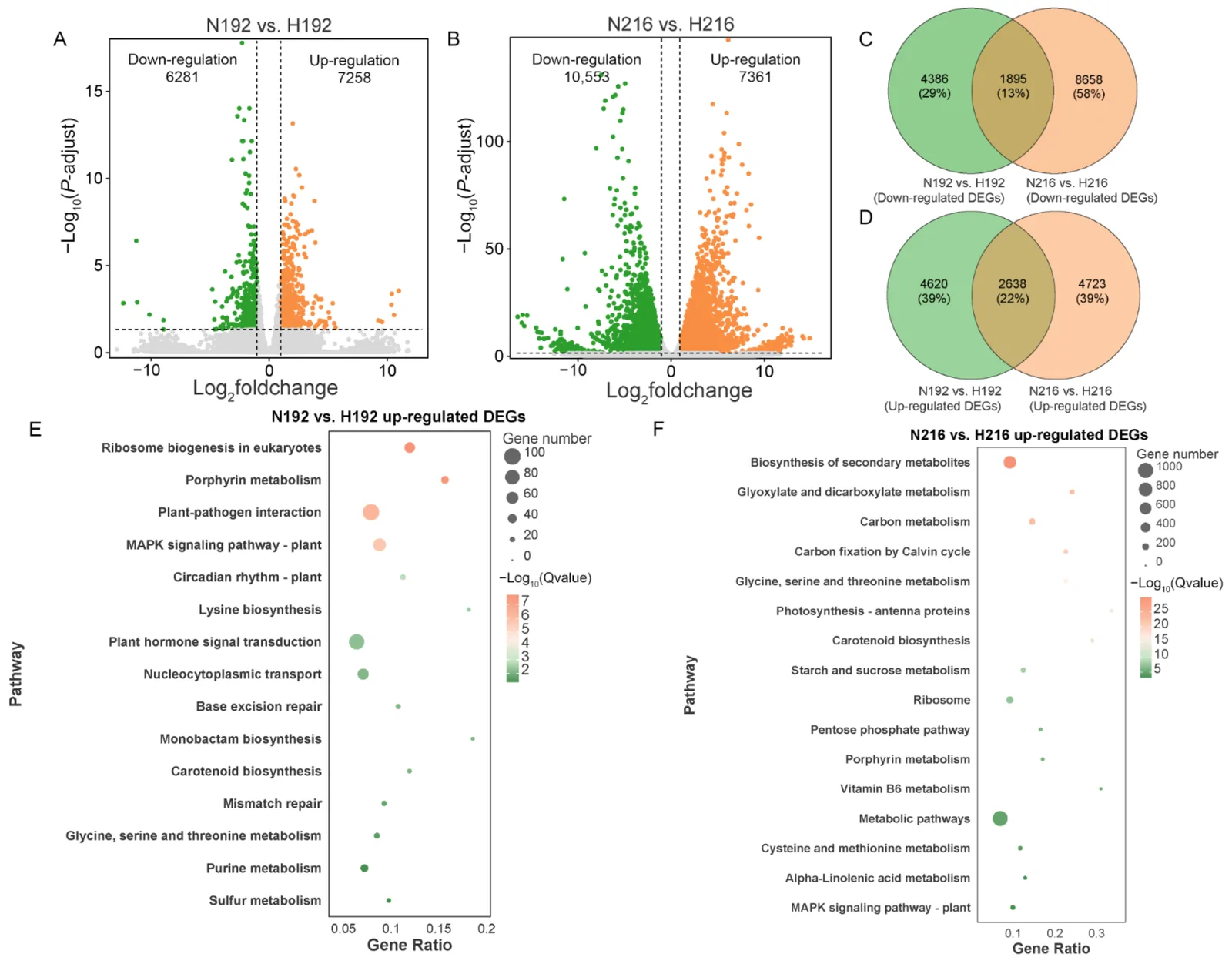
Transcriptome analysis of wheat inoculated with *Puccinia striiformis* f. sp. *tritici* at 192 and 216 hpi under normal and high temperatures. (**A**) Volcano plots of differential gene expression for N192 vs. H192 and (**B**) N216 vs. H216. Up- and down-regulated genes are labeled with orange and green dots, respectively. (**C**) Venn diagram of common down-regulated and (**D**) up-regulated gene expression between N192 vs. H192 and N216 vs. H216. (**E**) KEGG enrichment of up-regulated DEGs for N192 vs. H192 and (**F**) N216 vs. H216. The bubble plots show the enrichment of KEGG pathways with *Q*-value < 0.05. The abscissa represents the enrichment ratio, and the ordinate denotes the KEGG pathway. The size of bubbles indicates the number of genes annotated to a certain KEGG pathway, and the color represents the *Q*-value.

Compared to the NT treatment after inoculation with *Pst*, the number of DEGs under HT conditions exceeded 10,000 at each time point. Principal component analysis (PCA) showed separation between HT and NT samples at both sampling time points. PC1 and PC2 explained 39.8% and 30.1% of the total variance, respectively. Biological replicates of the inoculated groups generally clustered together, whereas the N216 samples showed greater dispersion (Figure S1). Differential expression analysis revealed that 13,539 and 17,914 DEGs were identified in the N192 vs. H192 and N216 vs. H216 comparisons, respectively. Among these, 6281 were down-regulated, and 7258 were up-regulated in N192 vs. H192, while 10,553 were down-regulated and 7361 were up-regulated in the N216 vs. H216 comparison (Figure 2A,B). Additionally, a total of 1895 down-regulated DEGs (13%) and 2638 up-regulated DEGs (22%) were identified as shared between the N192 vs. H192 and N216 vs. H216 (Figure 2C,D). Subsequent functional enrichment analysis of up-regulated DEGs using the KEGG database revealed that, at 192 hpi, these DEGs were primarily enriched in plant-pathogen interaction pathways, MAPK signaling pathways, plant hormone signal transduction pathways, and metabolism-related pathways (Figure 2E). In contrast, at 216 hpi, significant enrichment was observed in various metabolic pathways, including the biosynthesis of secondary metabolites, glyoxylate and dicarboxylate metabolism, carbon metabolism, glycine, serine and threonine metabolism, starch and sucrose metabolism, and cysteine and methionine metabolism (Figure 2F). At 216 hpi, the enriched carbohydrate, amino acid, and secondary metabolic pathways may support host defense by supplying energy and biosynthetic precursors and contributing to redox balance.

### 3.3. Co-Expression Networks Reveal Modules Associated with HTAS to Pst

**Figure 3:**
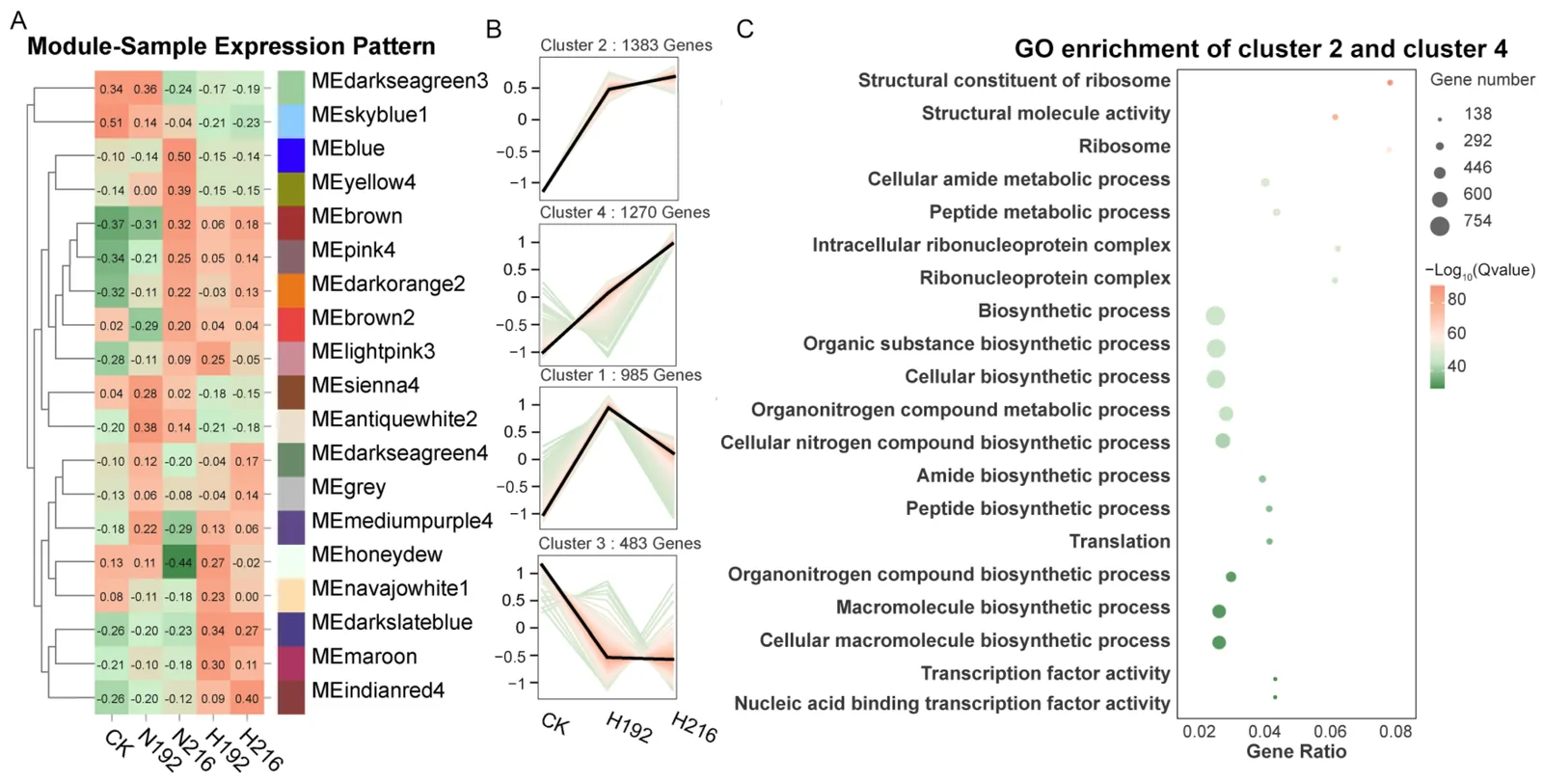
Weighted gene co-expression network analysis in N192, N216, H192, and H216. (**A**) Heatmap of module-trait relationships and gene expression patterns. (**B**) Analysis of gene expression trends in the H216-associated modules MEindianred4 and MEdarkslateblue. Each line represents one gene. (**C**) Gene Ontology (GO) terms enrichment analysis using Cluster 2 and Cluster 4.

To understand the dynamic changes in DEGs and identify modules related to HTAS to *Pst*, we performed WGCNA. A total of 19 co-expression modules were identified (Figure S2). Correlation analysis between module eigengenes (MEs) and gene expression profiles was performed to identify modules highly expressed under H216 treatment. The MEdarkslateblue and MEindianred4 modules were screened out, with ME values of 0.27 and 0.4, respectively (Figure 3A). We further performed trend analysis on these genes, which were clustered into four distinct groups based on their temporal expression patterns (Figure 3B). Among these, genes in cluster 2 and cluster 4 exhibited up-regulated expression over time under HT conditions and *Pst* infection. Furthermore, GO enrichment analysis revealed that the majority of the enriched GO terms were associated with the structural constituents of the ribosome and intracellular ribonucleoprotein complex (Figure 3C). These results indicate that the up-regulated clusters were enriched for ribosome-related genes in wheat following *Pst* infection under HT, suggesting that wheat rapidly reprograms its protein synthesis machinery as part of an enhanced defense response against pathogen attack.

### 3.4. Proteomic Analysis of DEPs and Functional Enrichment

**Figure 4:**
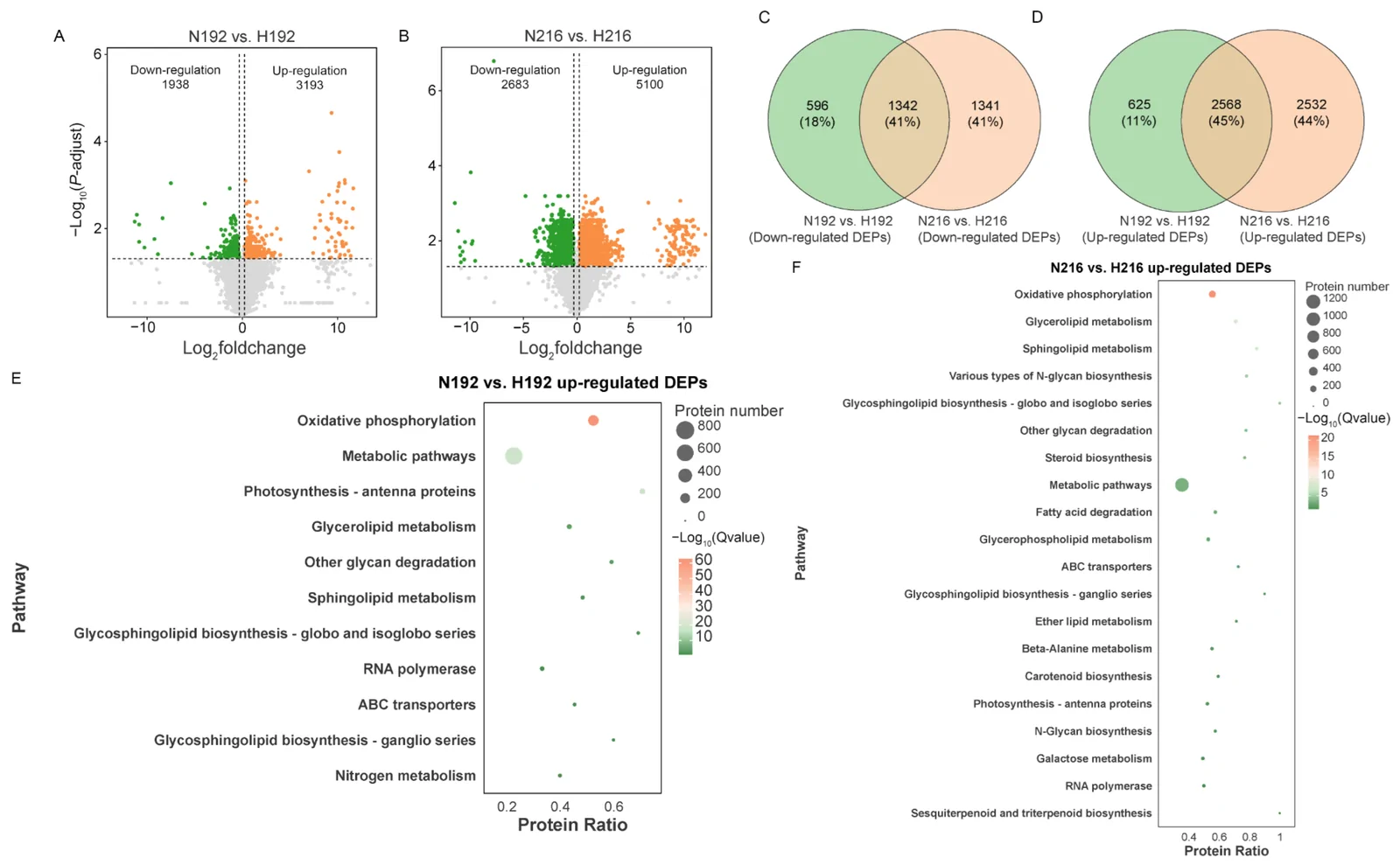
Proteomic analysis of wheat inoculated with *Puccinia striiformis* f. sp. *tritici* at 192 and 216 hpi under normal and high temperatures. (**A**) Volcano plots of differential protein expression for N192 vs. H192 and (**B**) N216 vs. H216. Up- and down-regulated proteins are labeled with orange and green dots, respectively. (**C**) Venn diagram of common down-regulated and (**D**) up-regulated protein expression between N192 vs. H192 and N216 vs. H216. (**E**) KEGG enrichment of up-regulated DEPs for N192 vs. H192 and (**F**) N216 vs. H216. The bubble plots show the enrichment of KEGG pathways with *Q*-value < 0.05. The abscissa represents the enrichment ratio, and the ordinate denotes the KEGG pathway. The size of bubbles indicates the number of proteins annotated to a certain KEGG pathway, and the color represents the *Q*-value.

Proteomics complements transcriptomic technologies in elucidating organismal adaptation mechanisms under stress. Proteomic profiling was conducted to assess the dynamics of wheat responses to *Pst* infection under NT and HT treatments. A total of 58,929 precursors, 53,978 peptides, and 14,888 proteins were obtained (Table S2). PCA was performed to assess sample relationships, revealing clear separation among samples from different treatments. PC1 and PC2 accounted for 63.6% and 14.0% of the total variance, respectively. Biological replicates of the inoculated groups clustered together, whereas CK samples exhibited greater dispersion (Figure S3). Comparative analysis identified 5131 DEPs in the N192 vs. H192 comparison, and 7783 DEPs in N216 vs. H216. Among these, 1938 were down-regulated and 3193 were up-regulated in the N192 vs. H192 comparison, while 2683 were down-regulated and 5100 were up-regulated in the N216 vs. H216 (Figure 4A,B). Furthermore, Venn analysis revealed 1342 commonly down-regulated (accounting for 41%) and 2568 commonly up-regulated DEPs (45%) between the two comparisons (Figure 4C,D), representing proportions substantially higher than those observed for DEGs.

Moreover, KEGG functional enrichment analysis of the up-regulated DEPs revealed distinct differences in pathway enrichment between 192 and 216 hpi (Figure 4E,F). In the N192 vs. H192 comparison, up-regulated DEPs were significantly enriched in pathways including oxidative phosphorylation, metabolic pathways, photosynthesis-antenna proteins, and ABC transporters. In contrast, DEPs in the N216 vs. H216 comparison were enriched in pathways such as various types of N-glycan biosynthesis, galactose metabolism, carotenoid biosynthesis, and triterpenoid biosynthesis.

### 3.5. Construction of Protein–Protein Interaction (PPI) Networks

**Figure 5:**
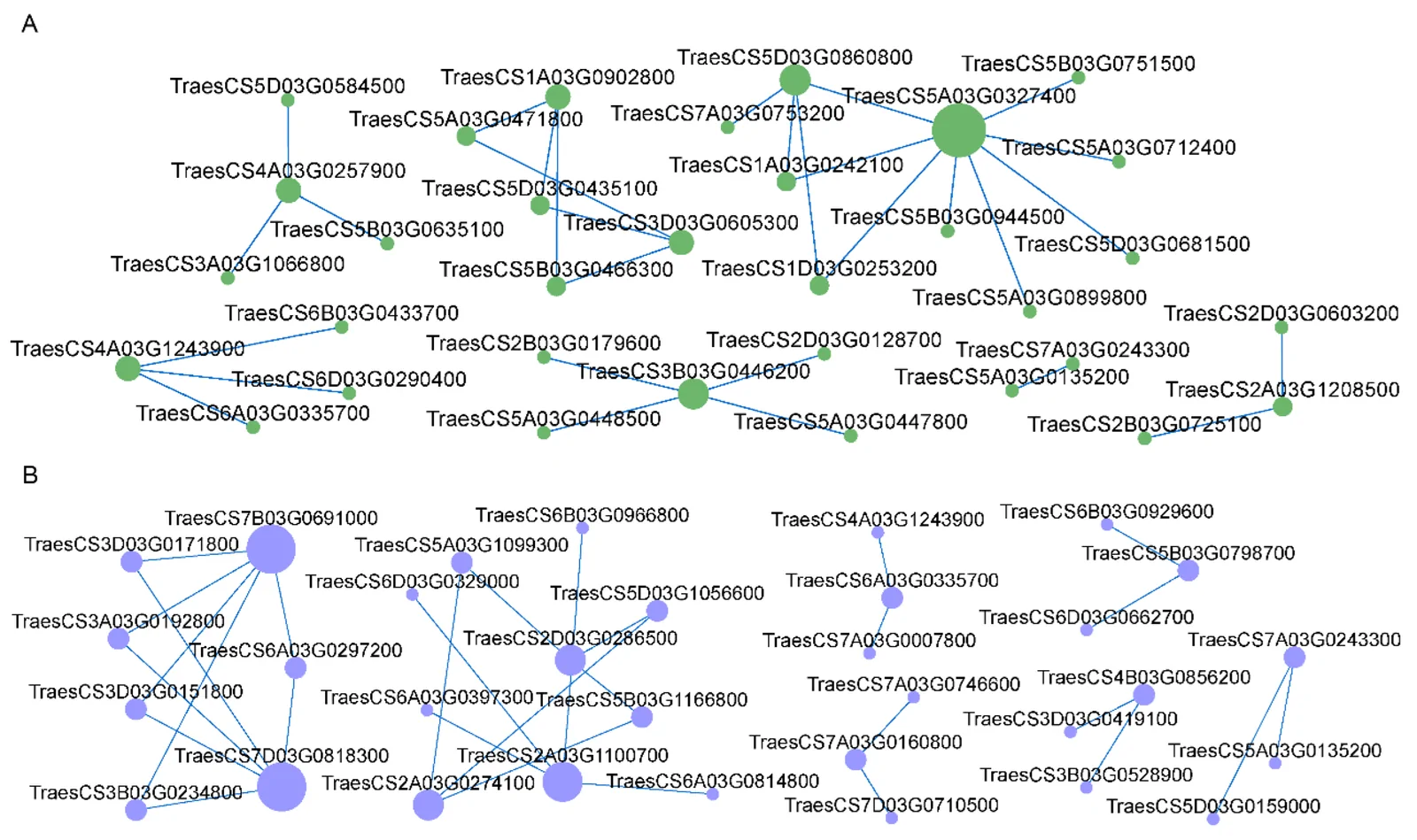
Protein–protein interaction (PPI) network. (**A**) PPI network diagram of differentially accumulated proteins from KEGG pathways (*Q*-value < 0.05) in the N192 vs. H192 comparison and (**B**) in the N216 vs. H216 comparison.

To elucidate the PPI networks underlying the wheat response to *Pst* infection under HT treatment, DEPs significantly enriched in KEGG pathways (*Q*-value < 0.05) were mapped to the STRING database to construct the corresponding PPI networks (Table S3). In the N192 vs. H192 comparison, the protein EMB3003 (*TraesCS5A03G0327400*) emerged as a hub protein (Figure 5A). This protein was predicted to interact with multiple targets, including proteins involved in diverse metabolic pathways, as well as the histone H1-like protein and a post-GPI attachment to proteins factor 3-like protein. In the N216 vs. H216 group, proteins LPLAT2 (*TraesCS7B03G0691000*) and RAB2A (*TraesCS7D03G0818300*) were identified as hub proteins. These hubs showed predicted associations not only with metabolic pathway components but also with proteins involved in transport and catabolism (*TraesCS3B03G0234800*) (Figure 5B). Functional annotations and evidence from homologous proteins link EMB3003 to acetyl-CoA production, LPLAT2 to phospholipid remodeling, and RAB2A to vesicle trafficking. These functions suggest potential contributions to HTAS through metabolic adjustment, membrane homeostasis, and defense-related transport, although their specific roles require functional validation.

### 3.6. Integrated Analysis of Transcriptome and Proteome

**Figure 6:**
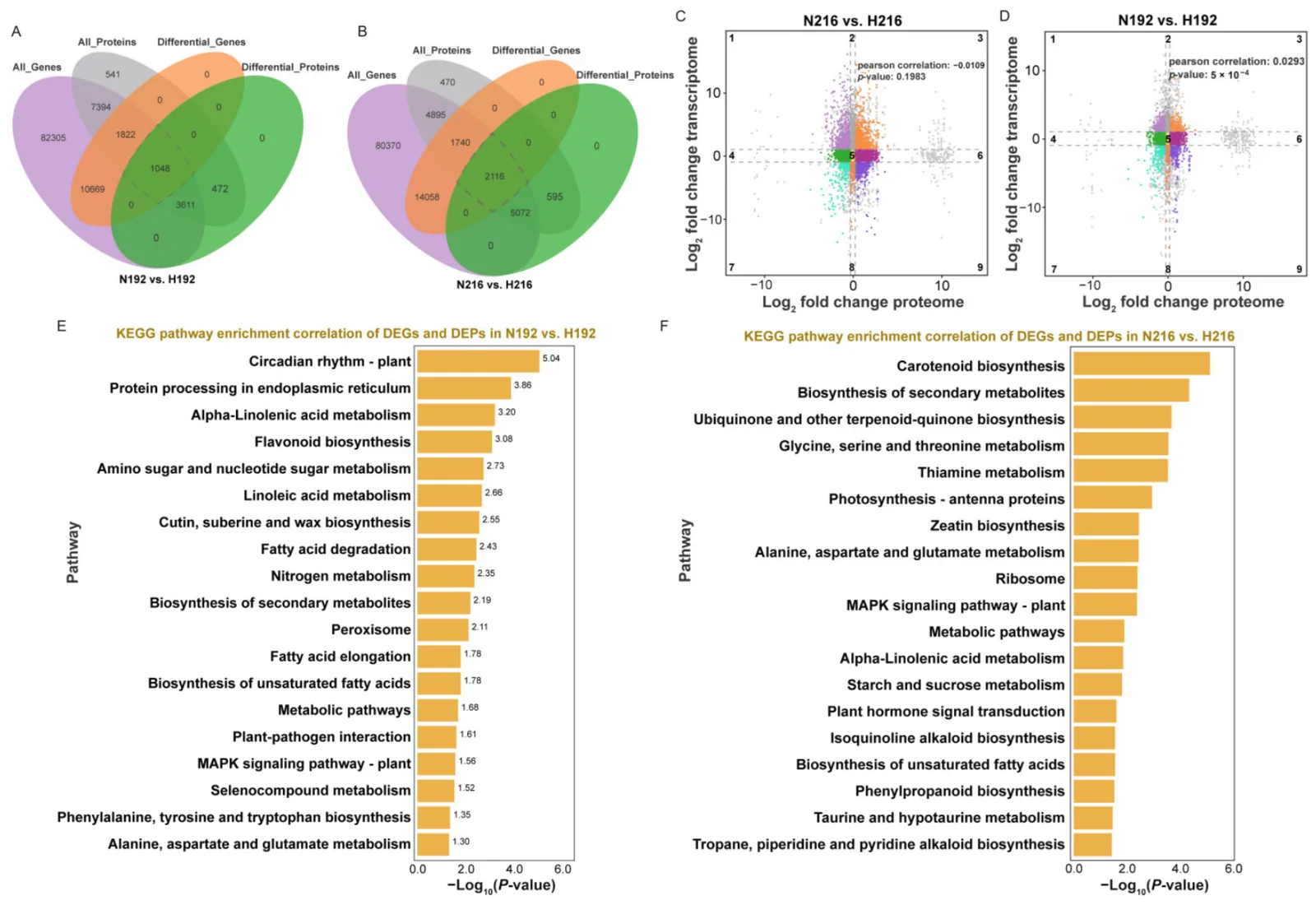
Correlations between mRNA and protein expression. (**A**) Venn diagram of genes quantified in the transcriptome (purple) and proteome (grey), differentially expressed genes (DEGs, orange), and differentially expressed proteins (DEPs, green) in N192 vs. H192 and (**B**) N216 vs. H216. (**C**) Scatterplot of the relationship between genes identified in both the transcriptome and proteome in N216 vs. H216 and (**D**) N192 vs. H192. (**E**) Combined enrichment of DEGs and DEP pathways in N192 vs. H192 and (**F**) N216 vs. H216.

To gain deeper insights into the HT resistance mechanism of the wheat landrace Paozimai, we integrated overlapping transcriptomic and proteomic data to elucidate the coordination between transcriptional and proteomic alterations. In the N192 vs. H192 group, a total of 13,875 genes or proteins were matched in the combined transcriptome and proteome analysis, accounting for 12.99% and 93.20% of the respective datasets. For the N216 vs. H216 comparison, 13,825 genes or proteins were matched through integrated analysis, corresponding to 12.77% and 92.85% of the transcriptomic and proteomic datasets, respectively. Moreover, integration of the proteome and transcriptome data showed that 1048 DEPs (192 hpi) and 2116 DEPs (216 hpi) were matched with their DEGs, respectively (Figure 6A,B). Overall, Pearson correlation analysis of fold changes revealed an extremely weak correlation between the proteome and transcriptome, with correlation coefficients of −0.0109 (*p* = 0.198) for the N216 vs. H216 comparison and 0.0293 (*p* = 5 × 10^−4^) for the N192 vs. H192 comparison (Figure 6C,D and Figure S4A,B). This weak correlation is consistent with regulation beyond transcript abundance, including translational and post-translational processes that affect protein abundance during the resistance response.

Combined analysis of DEGs and DEPs with consistent expression trends in the N192 vs. H192 group identified enrichment in circadian rhythm—plant, biosynthesis of secondary metabolites, metabolic pathways, flavonoid biosynthesis, MAPK signaling pathway-plant, and plant-pathogen interaction. In contrast, the N216 vs. H216 comparison showed significant enrichment in carotenoid biosynthesis, ubiquinone and terpenoid-quinone biosynthesis, photosynthesis-antenna proteins, zeatin biosynthesis, and plant hormone signal transduction (Figure 6E,F). These findings indicate that these pathways may be associated with wheat resistance to *Pst* infection under HT. Notably, at the key infection time point of 216 hpi, the responses were characterized by enrichment of genes and proteins associated with carotenoid biosynthesis, photosynthesis-related pathways, and hormone signaling.

### 3.7. Integrated Analysis Identifies Candidate Genes Associated with HTAS to Pst

**Figure 7:**
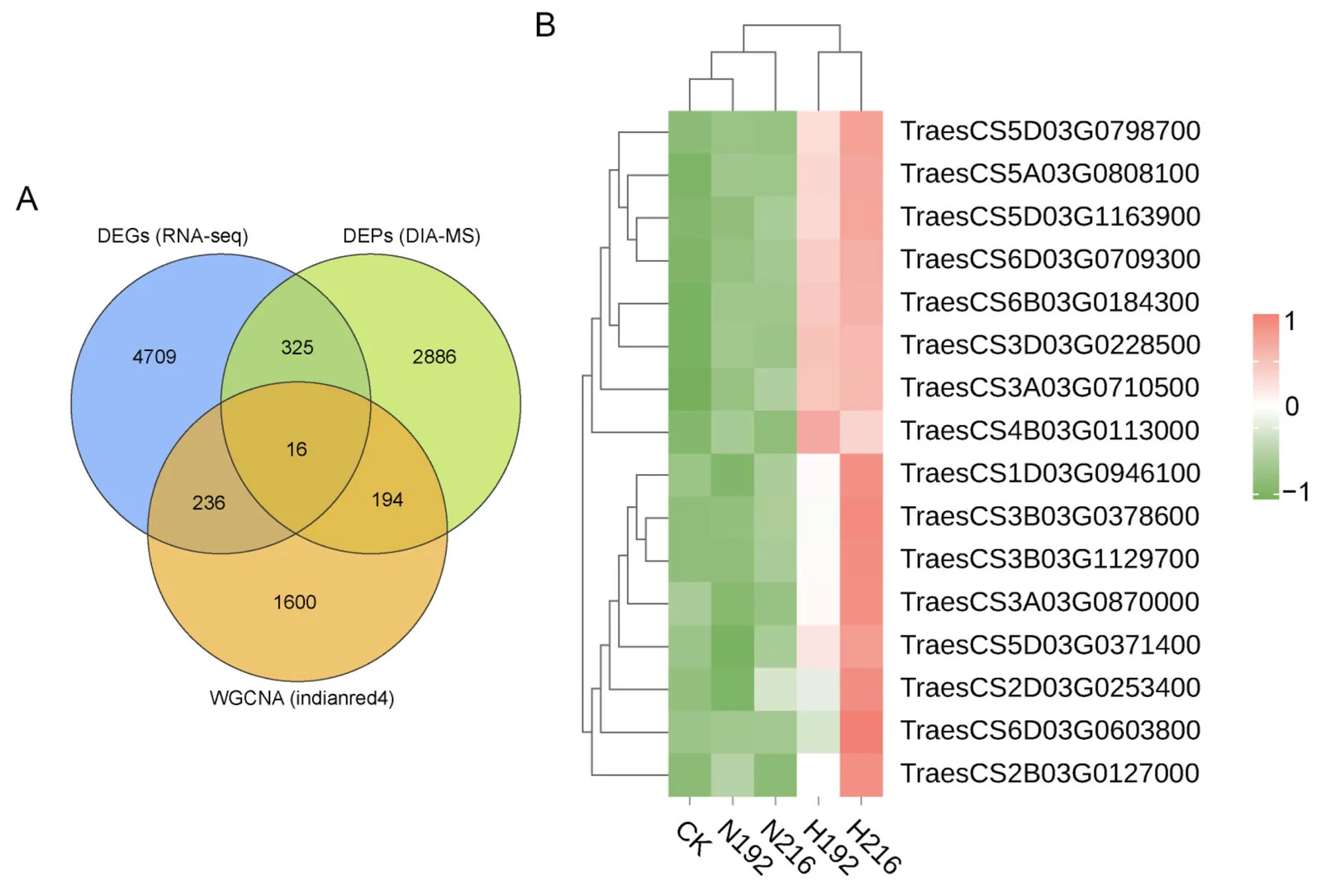
Identification and expression profiles of candidate genes associated with HTAS to *Pst* in wheat. (**A**) Venn diagram showing the overlap among transcriptomic DEGs, proteomic DEPs, and hub genes identified from the key regulatory modules via WGCNA. (**B**) Heat map showing the transcriptional expression profiles of candidate genes.

To further identify candidate genes associated with HTAS to *Pst* in the wheat landrace Paozimai, we integrated 5286 common DEGs and 3421 common DEPs shared between the 192 h and 216 h high-temperature treatments, together with 2046 genes from the indianred4 module that were highly expressed under H216 treatment as identified by WGCNA. Venn analysis of the intersection revealed a total of 16 candidate genes, all of which exhibited up-regulated transcriptional expression after both H192 and H216 treatments (Figure 7A,B). Functional annotation of these shared candidates revealed that the most prevalent category comprised genes encoding translation-associated proteins and proteins involved in RNA or protein homeostasis, including three ribosomal proteins (*TraesCS1D03G0946100*, *TraesCS3B03G0378600*, and *TraesCS5D03G0371400*), a glycine-rich RNA-binding protein 2 (*TraesCS3B03G1129700*), and a nucleolar protein (*TraesCS2D03G0253400*). The second major category encompassed metabolism-related proteins, such as a methyltransferase (*TraesCS2B03G0127000*), acyl-activating enzyme 16 (*TraesCS5D03G1163900*), and a clavaminate synthase-like protein (*TraesCS3D03G0228500*). In addition, we identified candidates involved in stress defense and chromatin regulation, including a temperature-induced lipocalin-1 (*TraesCS6D03G0709300*) and two histone H2B variants (*TraesCS5D03G0798700* and *TraesCS6B03G0184300*) (Table S4). However, their potential roles in HTAS require further functional validation.

## 4. Discussion

Previous phenotypic studies of the wheat landrace Paozimai reported its resistance to *Pst* under high-temperature conditions. Compared to NT treatment, HT treatment triggered reactive oxygen species (ROS) production, restricted hyphal expansion, and ultimately led to a reduction in the number of uredinia produced [20,28]. Based on previous histological and phenotypic evaluations of wheat-*Pst* interactions, the period around 192 and 216 hpi represents the late latent period where macroscopic resistance phenotypes, such as the formation of necrotic lesions and the restriction of fungal proliferation, become highly pronounced between NT and HT conditions [12]. This study characterized the molecular basis of HTAS to *Pst* in Paozimai at 192 and 216 hpi through integrated transcriptomic and proteomic profiling. The transcriptome showed a stage-dependent shift: at 192 hpi, up-regulated DEGs are enriched in plant–pathogen interaction, MAPK signaling, and hormone signal transduction (Figure 2E), whereas at 216 hpi, metabolic pathways become dominant (Figure 2F). These enrichment patterns are consistent with a shift from signaling-related to metabolism-related processes between 192 and 216 hpi. Such sustained metabolic changes may help restrict biotrophic pathogens and maintain defense outputs long after the initial signaling burst [37,38]. In wheat HTAS resistance contexts, signaling-related responses have also been reported [28], consistent with the pathway enrichment observed at 192 hpi.

Functional enrichment analysis of *Pst* up-regulated DEGs highlighted protein processing and fatty acid metabolism at 192 hpi and carbohydrate and glutathione metabolism at 216 hpi, indicating differences in the functional categories represented by up-regulated genes between the two sampling time points. Although fungal growth is largely restricted under high temperature, specific metabolic genes are up-regulated. In carbohydrate metabolism, *PSTG_11670* (α-glucosidase) was up-regulated at 216 hpi and may contribute to sugar mobilization to sustain basal energy needs [39]. Furthermore, *PSTG_11438* (chitin deacetylase) and *PSTG_00650* (tyrosinase) were markedly up-regulated, which may respectively mediate cell-wall chitin-to-chitosan conversion to evade host chitinases and promote melanin biosynthesis for ROS scavenging [40,41]. These findings are consistent with previous reports showing that temperature can reshape the central carbon and energy metabolic landscapes of *Pst* [9,39]. These changes may reflect fungal responses to the altered temperature, changes in the infected host, or both.

The enrichment of cysteine and methionine metabolism is particularly noteworthy because sulfur-containing metabolism is intimately tied to redox buffering, glutathione-related processes, and broader ROS homeostasis, which are central to limiting biotroph proliferation without incurring runaway host damage [42,43]. Previous studies showed that *Pst* effectors can target ROS-related processes to suppress host resistance [44,45,46], implying that the metabolic rerouting may represent a countermeasure that reinforces ROS scavenging capacity and preserves cellular integrity under combined heat and pathogen stress. This interpretation also aligns with the broader view that durable defense, especially against biotrophic pathogens, requires continuous metabolic support for defense compound synthesis and homeostasis maintenance rather than transient transcriptional activation alone [37,47]. The WGCNA further clarifies the regulatory architecture of HTAS by identifying the MEdarkslateblue and MEindianred4 as co-expression modules, with genes in clusters 2 and 4 showing sustained up-regulation under HT treatment and *Pst* infection (Figure 3A,B). GO enrichment indicates that these clusters are dominated by terms related to ribosome structure and protein biosynthesis (Figure 3C), highlighting de novo protein synthesis capacity as a candidate regulatory component of the HTAS resistance program. This result complements previous work, which showed that the CRK–RLCK–MAPKs module regulates HTAS resistance in wheat cv. XY6 via protein expression, and it extends foundational perspectives that effective immunity depends on timely accumulation of defense-related proteins [16]. Sustained protein synthesis may help replenish receptors, kinases, and PR proteins during the prolonged infection cycle typical of biotrophic *Pst*. In this framework, the contribution of trafficking and delivery becomes equally important. TaRABH1bL, a small GTPase implicated in protein trafficking during HTAS resistance, supports the idea that wheat coordinates protein synthesis with protein transport to maintain functional defense deployment at infection interfaces [17].

Proteomic analysis revealed a distinguishing feature of HTAS relative to conventional race-specific ASR: 41% of down-regulated and 45% of up-regulated DEPs are shared between 192 hpi and 216 hpi (Figure 4C,D), far exceeding the shared DEG ratio (Figure 2C,D). This comparatively stable proteomic response could potentially contribute to the durability of HTAS, because sustained defense protein availability reduces reliance on narrowly timed transcriptional spikes and may lower the probability of resistance breakdown typical of ASR under rapidly evolving *Pst* populations [11]. DEP enrichment also supports functional partitioning across time. Oxidative phosphorylation at 192 hpi may contribute to ATP production, whereas N-glycan biosynthesis at 216 hpi suggests a role for glycosylation in tuning immune protein localization and activity late in the response. N-glycosylation and secretory pathway quality control are crucial for the maturation, stability, and function of RLKs [48,49]. Thus, enrichment of glycosylation-related pathways at 216 hpi could represent a regulatory layer that maintains immune competence under high temperature when protein folding and membrane trafficking are particularly challenged.

The integrated analysis revealed weak overall correlations between transcript and protein fold changes (Figure 6C,D). This discrepancy may reflect several factors, including differences in mRNA and protein stability, variation in translation efficiency, and a time lag between transcriptional changes and protein accumulation [50,51]. The consistency observed in DEG–DEP pathway sets suggests that a subset of pathways functions as regulatory nodes whose coordination is preserved across expression layers, anchoring resistance while other processes are more flexibly regulated post-transcriptionally. In hormone-mediated defense, cytokinins have been reported to enhance resistance to biotrophic pathogens by modulating immune signaling networks, providing a plausible mechanistic bridge between enriched hormone-related signals and defense responses during the late latent period [52]. In addition, the presence of ABC transporter enrichment in proteomic and integrated analyses suggests that transport-related processes may be associated with HTAS, consistent with the finding that *Yr18*, a key gene encoding an ABC transporter, confers HTAP resistance in wheat [13]. The ABC transporter function is conserved across temperature-dependent resistance types and underscores their universal importance in defense-associated export and nonhost resistance processes [53]. Together, these findings point to membrane transport as a potentially shared component of wheat rust defense, although the relationship between the specific transporters identified here and *Yr18* remains to be determined. This study identified transcriptomic and proteomic changes associated with HTAS in Paozimai and highlighted 16 candidate wheat genes. However, the interpretation of these findings relies mainly on bioinformatics analyses, including functional enrichment and network analysis, without direct physiological or metabolic measurements to support the proposed biological processes. Further functional and genetic validation is needed to clarify the roles of the candidate genes in HTAS resistance. Interpretation of the temperature comparisons is further constrained by the absence of non-inoculated plants maintained under the same normal- and high-temperature regimes and sampled at the corresponding time points. Thus, the observed HT–NT differences may include direct responses of wheat to temperature and cannot be attributed solely to changes in the *Pst*–wheat interaction.

## 5. Conclusions

In this study, integrated dual RNA-seq and DIA proteomic analyses characterized the responses of wheat landrace Paozimai and *Pst* to contrasting temperatures at 192 and 216 hpi, two time points in the late latent period. Up-regulated *Pst* genes showed different patterns of pathway enrichment across the two time points. In infected wheat, DEGs were enriched in defense-related signaling pathways at 192 hpi and metabolism-related pathways at 216 hpi. A greater proportion of wheat DEPs than DEGs was shared between the two time points, while co-expression analysis highlighted ribosome-related processes. Integrated analysis identified 16 candidate wheat genes associated with HTAS. These findings provide a basis for investigating the molecular responses of wheat and *Pst* under different temperatures. The functions of the enriched pathways and candidate genes remain to be established. Future physiological, metabolic, and genetic studies should clarify their contributions to HTAS resistance.

## Data Availability

The data presented in this study are available within the article and the Supplementary Materials. The RNA-seq data generated in this study have been deposited at NCBI under BioProject accession PRJNA1535521.

## Supplementary Materials

The following supporting information can be downloaded at https://www.mdpi.com/article/10.3390/jof12100765/s1, Figure S1. Principal component analysis (PCA) of RNA-seq data across different treatments; Figure S2. Gene cluster dendrogram and module identification in WGCNA; Figure S3. PCA of proteomic data across different treatments; Figure S4. Differentially expressed genes (DEGs) and differentially expressed proteins (DEPs) expression correlations. (A) Scatterplot and correlation coefficients between DEGs and DEPs (the same trend) in N192 vs. H192 and (B) N216 vs. H216. Table S1. Data filtering and quality control statistics; Table S2. Statistical table of protein and peptide identification results; Table S3. Protein-protein interaction (PPI) network; Table S4. Functional annotation of candidate genes.

## Abbreviations

The following abbreviations are used in this manuscript:

ABC	ATP-binding cassette
APR	adult-plant resistance
ASR	all-stage resistance
BCA	bicinchoninic acid
DEG	differentially expressed gene
DEP	differentially expressed protein
DIA	data-independent acquisition
FDR	false discovery rate
GC	guanine–cytosine
GFF	general feature format
GO	Gene Ontology
hpi	hours post-inoculation
HT	high temperature
HTAP	high-temperature adult-plant
HTAS	high-temperature all-stage
KEGG	Kyoto Encyclopedia of Genes and Genomes
NT	normal temperature
PCA	principal component analysis
PPI	protein–protein interaction
PR	pathogenesis-related
*Pst*	*Puccinia striiformis* f. sp. *tritici*
ROS	reactive oxygen species
STRING	Search Tool for the Retrieval of Interacting Genes/Proteins
TPM	transcripts per million
WGCNA	Weighted gene co-expression network analysis
