# Supplementary material for: Integrated Transcriptomic and Proteomic Analyses Reveal Wheat Responses Associated with High-Temperature All-Stage Resistance to Puccinia striiformis f. sp. tritici
Source: J Fungi (Basel). 2026 Oct 11;12(10):765. doi: 10.3390/jof12100765 (PMC13706213; doi:10.3390/jof12100765)
Supplement: Supplementary file 1 [file jof-12-00765-s001.zip › Supplemental Figures.pdf]

Supplemental Materials

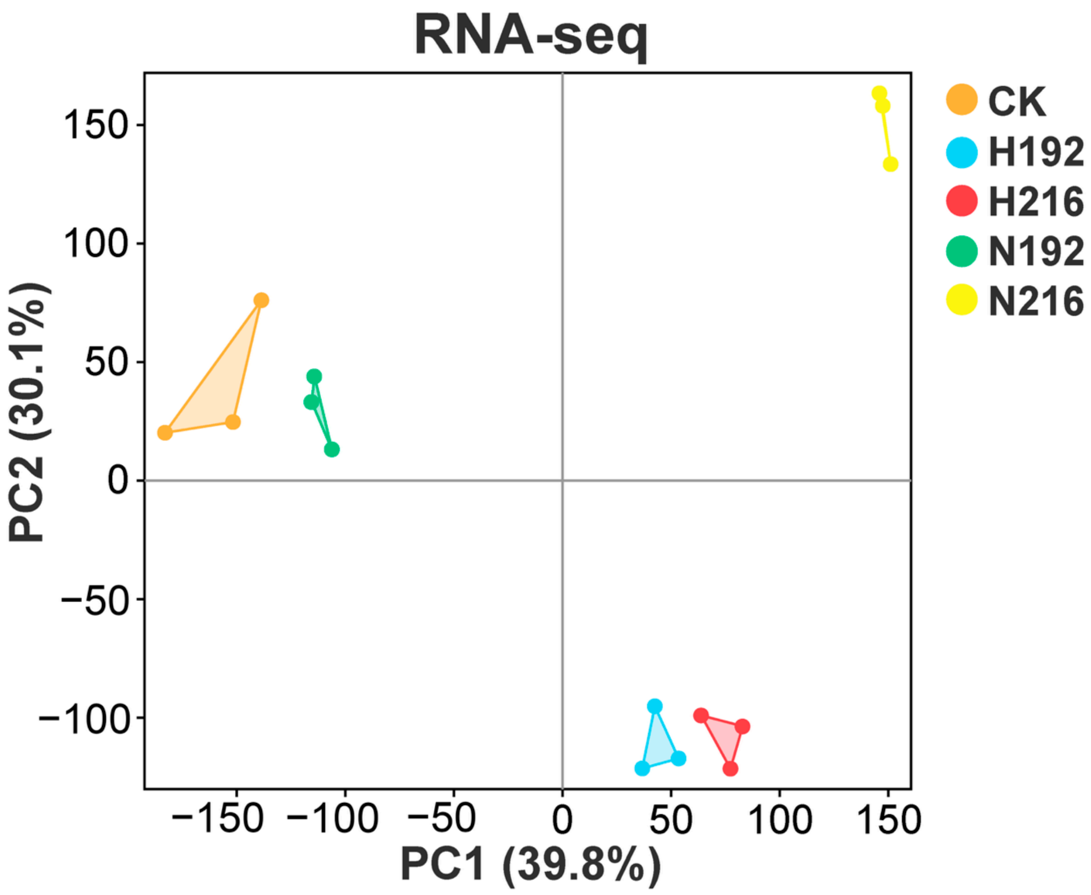

Figure S1. Principal component analysis (PCA) of RNA-Seq data across different treatments. Each point represents an independent biological replicate (n = 3 per group). The samples are color-coded as follows: CK (orange), H192 (light blue), H216 (red), N192 (green), and N216 (yellow).

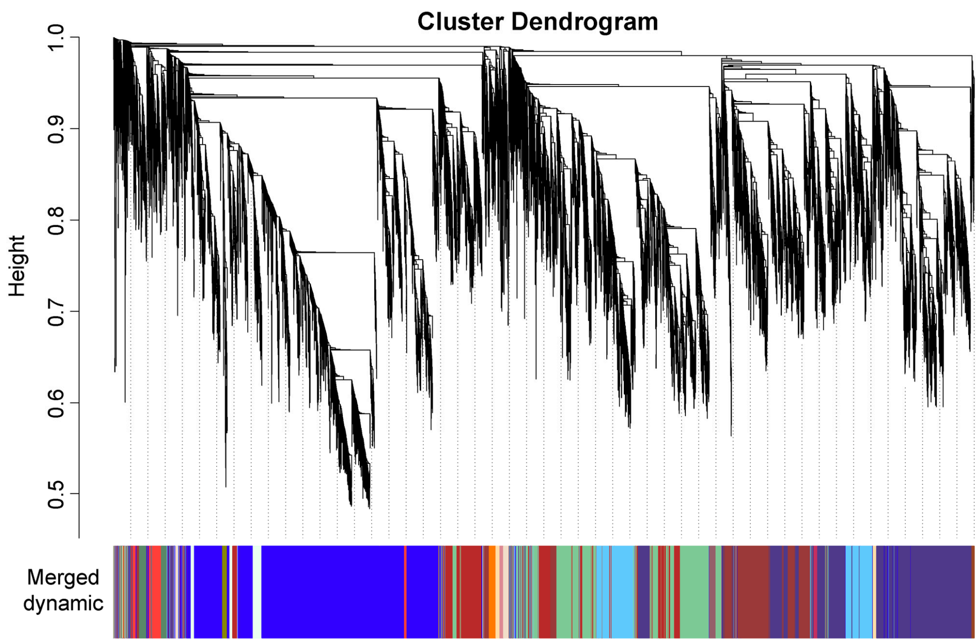

Figure S2. Gene cluster dendrogram and module identification in WGCNA analysis

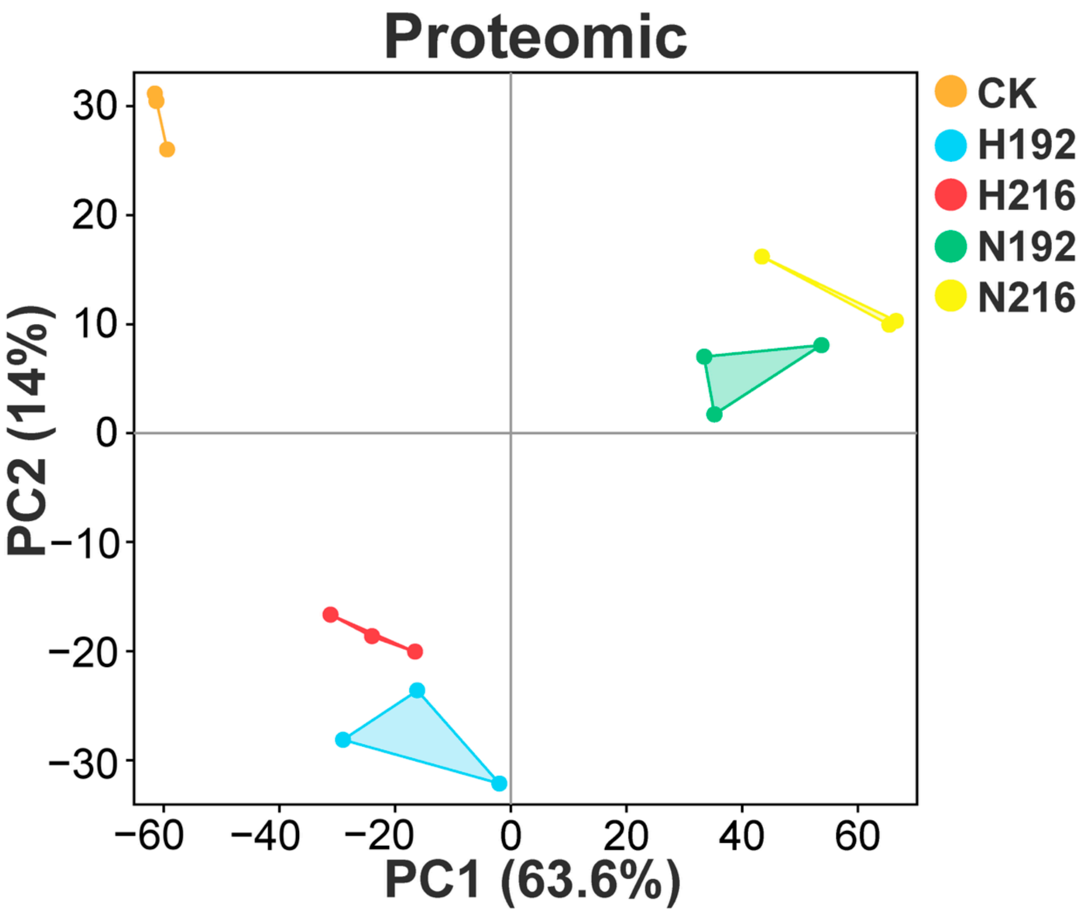

Figure S3. PCA of proteomic data across different treatments. Each point represents an independent biological replicate (n = 3 per group). The samples are color-coded as follows: CK (orange), H192 (light blue), H216 (red), N192 (green), and N216 (yellow).

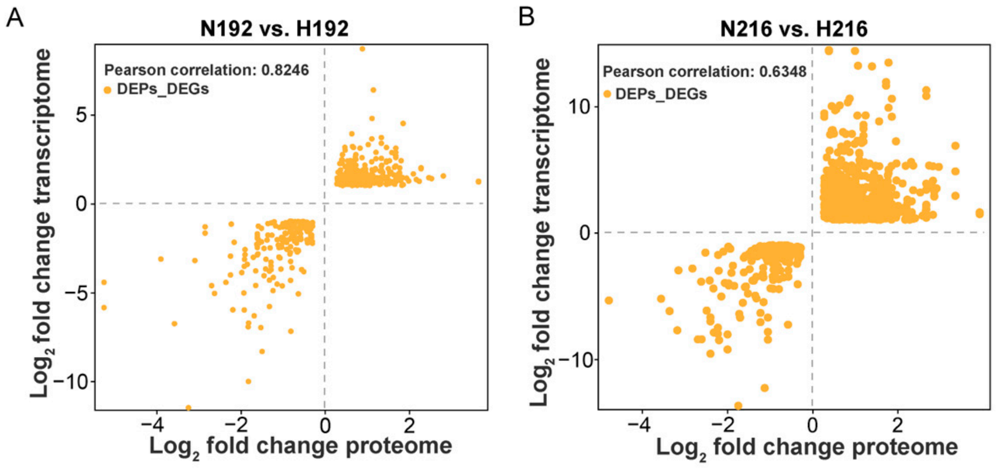

Figure S4. Differentially expressed genes (DEGs) and differentially expressed proteins (DEPs) expression correlations. (A) Scatterplot and correlation coefficients between DEGs and DEPs (the same trend) in N192 vs. H192 and (B) N216 vs. H216.
